# Early prediction of cardiovascular events following treatments in female breast cancer patients: Application of real-world data and artificial intelligence

**DOI:** 10.1016/j.breast.2025.104438

**Published:** 2025-03-10

**Authors:** Quynh T.N. Nguyen, Shwu-Jiuan Lin, Phung-Anh Nguyen, Phan Thanh Phuc, Min-Huei Hsu, Chun-Yao Huang, Chin-Sheng Hung, Christine Y. Lu, Jason C. Hsu

**Affiliations:** a School of Pharmacy, College of Pharmacy, Taipei Medical University, Taipei City, Taiwan; b Institute of Pharmaceutical Education and Research, Binh Duong University, Binh Duong province, Viet Nam; c Graduate Institute of Data Science, College of Management, Taipei Medical University, Taipei, Taiwan; d Clinical Data Center, Office of Data Science, Taipei Medical University, Taipei City, Taiwan; e Clinical Big Data Research Center, Taipei Medical University Hospital, Taipei Medical University, Taipei City, Taiwan; f Research Center of Health Care Industry Data Science, College of Management, Taipei Medical University, Taipei City, Taiwan; g International Ph.D. Program in Biotech and Healthcare Management, College of Management, Taipei Medical University, Taipei City, Taiwan; h Division of Cardiology, School of Medicine, College of Medicine, Taipei Medical University, Taipei City, Taiwan; i Department of Surgery, Taipei Medical University Hospital, Taipei Medical University, Taipei City, Taiwan; j Department of Population Medicine, Harvard Medical School and Harvard Pilgrim Health Care Institute, Boston, MA, USA; k Kolling Institute, Faculty of Medicine and Health, The University of Sydney and the Northern Sydney Local Health District, Sydney, NSW, Australia; l School of Pharmacy, Faculty of Medicine and Health, The University of Sydney, Sydney, New South Wales, Australia

## Abstract

• Application of real-world data and artificial intelligence in detecting cardiotoxicity following cancer treatment.
• Clinical features have been used to develop prediction models.
• Important features include age, tumor size, hypertension, HbA1c, HDL, creatinine, bilirubin, BUN, ALT, and diabetes.
• This study offers potential approaches for cardio-oncology clinical practice.

## 1 Introduction

Although breast cancer is not common in males, it is the leading cancer for both sexes combined [1]. Nonetheless, the 5-year survival rate among breast cancer patients is quite high, reaching 90 % [2]. The global count of cancer survivors is on the rise, emerging as a rapidly expanding group within the healthcare systems of numerous countries [3,4]. Considering many shared risk factors between cardiovascular disease and cancer (such as obesity, diabetes, smoking, and alcohol consumption) [5], the population of cancer patients is confronted with a heightened risk of cardiac issues.

Another significant concern is the association between cancer treatment and cardiac disease. Cardiotoxic effects of anticancer medication were initially identified in the 1960s. For instance, anthracyclines can lead to cardiac failure, while antimetabolites carry a high risk of heart attack [6,7]. Many epidemiological studies have also found an association between new anticancer drugs (such as targeted therapy and immune therapy) and cardiovascular events [[8], [9], [10], [11], [12]]. With the advancement of anticancer drugs, other heart-related ADRs have emerged and attracted the attention of health authorities, pharmaceutical companies, and clinicians.

Cardiovascular complications can interrupt cancer treatment and significantly impact life quality of cancer survivors. To predict such adverse events, researchers have conducted machine learning studies. Most models are based on physiochemical properties, making them more suitable for drug discovery and development [13,14]. Some studies have considered clinical features and the implementation in clinical practice. However, they have explored general cancer populations [15,16]. To our knowledge, machine learning models developed for predicting cardiovascular events in breast cancer patients is limited.

In this study, we developed a computational model to predict cardiovascular events following systemic therapy in female patients who suffered from breast cancer. We aimed to evaluate model performance and identify important features for predicting the outcomes.

## 2 Methods

### 2.1 Data source

This is a retrospective study utilizing data from Taipei Medical University Clinical Research Database (TMUCRD) [17]. TMUCRD obtains data from electronic health records of three affiliated hospitals. This database includes data from 3.8 million individuals in the period between 1998 and 2020, comprising basic information, comorbidities, medication use, laboratory test results, semi-structured, and unstructured data. TMUCRD is linked to Taiwan Cancer Registry (TCR) which provides details of cancer characteristics such as stage, tumor size, lymph node, specific biomarkers, etc. Prior to analysis, this study received approval from Taipei Medical University Joint Institute Review Board. The data was anonymized to ensure privacy.

### 2.2 Cohort selection

In the current study, female patients confirmed with primary breast cancer (ICD-O-3 code: C50) from 2004 to 2020 were selected. Exclusion criteria were age under 18 years old, absence of medical history during observational period (1 year prior to diagnosis date), and no record of chemotherapy or targeted therapy administration on the database.

### 2.3 Outcome definition

The outcome was defined by utilizing electronic health records from TMUCRD. The focus of our investigation was cardiovascular events, which comprised arrhythmia, myocardial infarction (MI), conduction disorders, coronary artery diseases (CAD), heart failure (HF), and stroke. The index date was determined as the date when patients were prescribed chemo and/or targeted agents. The outcome was the occurrence of any cardiovascular events during the follow-up time (1 year since the index date). To identify these outcomes, we extracted data using *International Classification of Diseases 9 and 10* from inpatient and outpatient records. The outcome included newly diagnosed cardiovascular events and cardiovascular events that required hospitalization. Details of the ICD code for six outcomes are listed in Supplementary materials.

### 2.4 Feature selection

This study has incorporated many clinical features which consisted of.

1. Demographic information: well-known risk factors for cardiac disease include age, tobacco and alcohol consumption, body mass index (BMI).
2. Cancer-related factors: cancer stage, tumor size, specific biomarkers of breast cancer (e.g., human epidermal growth factor receptor 2, hormone status), and cancer treatments (e.g., radiation, surgery).
3. Comorbidities: hyperlipidemia, hypertension, diabetes, liver disease, kidney disease, cerebrovascular disease, chronic pulmonary disease, and pre-existing heart conditions are considered conditions that may favor the development of cardiac events according to literature review [[18], [19], [20], [21], [22]]. Utilizing the power of machine learning to explore new predictors, our prediction model also incorporated other diseases from Charlson comorbidities index.
4. Concurrent medications: top 10 frequently used medications in the cohort were selected, including statins, metformin and other biguanides, calcium channel blockers (CCBs), beta-blockers, angiotensin II receptor blockers (ARBs), benzodiazepines, antiplatelets, sulfonylureas, DPP-4 inhibitors, and coxibs.
5. Laboratory tests: cardiac biomarkers (Troponin T/I, BNP/pro-BNP), left ventricular ejection fraction (LVEF), and other routine lab tests.

For the assessment of comorbidities, we evaluated those diagnosed during one year prior to the index date. Pre-existing cardiac disease is defined as any cardiac event diagnosed before the index date. Regarding medication usage, we considered patients who had received the medications for more than one month within the year prior to the index date.

### 2.5 Model development and evaluation

The features were tested on 8 machine learning models: Logistic Regression, Linear Discriminant Analysis, Bagging Classifier, Gradient Boosting Classifier, Random Forest Classifier, Light Gradient Boosting Machine Classifier, Extreme Gradient Boosting Classifier, and Voting Classifier. Voting Classifier is an ensemble technique that combines various models to predict an outcome. In this study, Voting Classifier was an ensemble of LDA and RF. The outcome was predicted based on the average probability given to it (soft voting).

The training dataset consisted of data from two hospitals (Taipei Medical University and Wan-Fang hospitals). To evaluate general performance and optimize hyperparameters for the machine learning algorithms, we employed stratified 5-fold cross-validation on the training dataset. For external testing and model generalization, data from Shuang-ho hospital was utilized as the external testing dataset.

To analyze the contribution of features to the best model, we utilized SHAP values (Shapley Additive explanation). Various metrics were employed to evaluate the model's performance, encompassing area under the receiver operating characteristic curve (AUROC or AUC), accuracy, sensitivity (recall), specificity, positive predictive value (precision or PPV), negative predictive value (NPV), and F1-score.

MSSQL Server 2017 was used to process data. Models were trained and tested using Python version 3.8.

## 3 Results

### 3.1 Overview of the study population

**Fig. 1:**
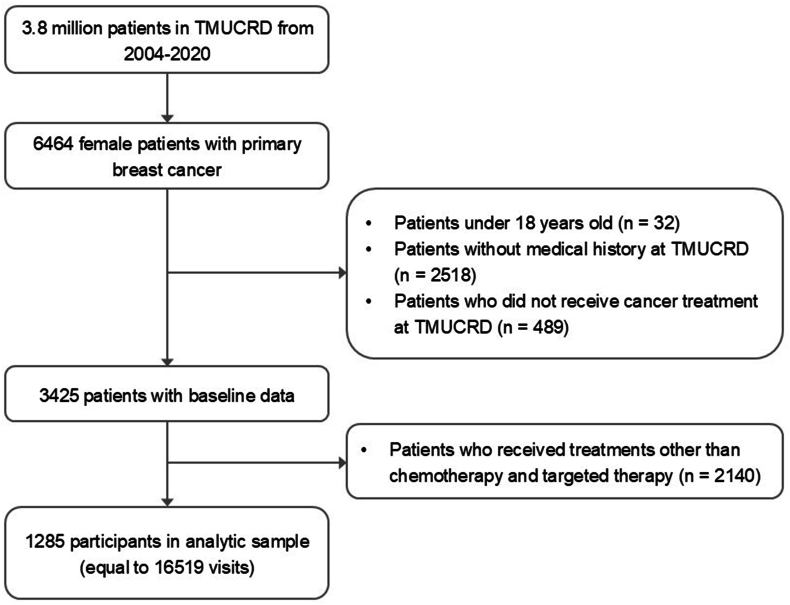
Cohort selection process.

We found 6464 females who were newly diagnosed with breast cancer and registered in the TCR. 3039 patients were excluded, comprising those younger than 18 years old, individuals lacking medical history on the database, and subjects who did not undergo cancer treatment at the affiliated hospitals. Eventually, 1285 individuals who received chemotherapy or targeted agents were included in the study. This dataset comprised 16,519 visits, with 9015 visits allocated to the training data and 7504 visits to the testing data (Fig. 1).

The baseline characteristics of the studied population are presented in Table 1. The mean (SD) age and BMI were 56.1 years (11.5 years) and 24.3 (4.0), respectively. Most patients were diagnosed with early stages: stage 0 (3.7 %), stage I (27.8 %), and stage II (49.5 %). They were less likely to drink (5.4 %) or smoke (7.3 %). A majority of the cohort underwent surgery (91.7 %) and/or radiation therapy (62.4 %) as first course treatment. Among the population, 97.9 % were treated with chemotherapy, 6.8 % were administered with targeted therapy. Hypertension (22.6 %), hyperlipidemia (17.9 %), and diabetes (14.2 %) were the three most common comorbidities in the cohort. Furthermore, 241 (18.8 %) patients had pre-existing cardiac disease. Within 1 year since the index date, 36 (2.8 %) patients experienced severe cardiovascular events, including 18 newly diagnosed cases and 18 cases with a history of heart disease requiring hospitalization. Coronary artery disease was the most common type (n = 14), followed by arrhythmia (n = 12), heart failure (n = 9), and stroke (n = 7). Only one case of conduction disorder was reported, while no myocardial infarction occurred during the follow-up period.

**Table 1:** Characteristics of the study population.

	**Overall (n=1285)**	**Training cohort**^a^**(n=703)**	**Testing cohort**^b^**(n=582)**
**Cardiac outcomes, N (%)**	36 (2.8)	22 (3.1)	14 (2.4)
Arrhythmia	12 (0.9)	9 (1.3)	3 (0.5)
CAD	14 (1.1)	10 (1.4)	4 (0.7)
HF	9 (0.7)	4 (0.6)	5 (0.9)
Conduction disorder	1 (0.1)	1 (0.1)	0 (0)
Myocardial infarction	0 (0)	0 (0)	0 (0.0)
Stroke	7 (0.5)	3 (0.4)	4 (0.7)
Newly diagnosed cardiac events	18 (1.4)	8 (1.1)	10 (1.7)
**Demographic information**			
Age, Mean (SD), yrs.	56.1 (11.5)	56.6 (12.1)	55.5 (10.7)
BMI, Mean (SD), kg/m^2^	24.3 (4.0)	24.1 (4.0)	24.6 (4.1)
Smoking, N (%)	94 (7.3)	46 (6.5)	48 (8.2)
Drinking, N (%)	69 (5.4)	45 (6.4)	24 (4.1)
**Cancer condition**			
Tumor size, mm			
Mean (SD)	29.3 (20.0)	29.6 (21.1)	28.9 (18.4)
Median [IQR]	24.0 [16.0, 35.0]	24.0 [16.0, 35.0]	24.0 [17.0, 35.0]
Cancer stage, N (%)			
stage = 0	47 (3.7)	29 (4.1)	18 (3.1)
stage = 1	357 (27.8)	214 (30.4)	143 (24.6)
stage = 2	636 (49.5)	355 (50.5)	281 (48.3)
stage = 3	78 (6.1)	46 (6.5)	32 (5.5)
stage = 4	96 (7.5)	50 (7.1)	46 (7.9)
Unknown	71 (5.5)	9 (1.3)	62 (10.7)
HER2, N (%)			
Negative	820 (63.8)	447 (63.6)	373 (64.1)
Positive	358 (27.9)	193 (27.5)	165 (28.4)
Unknown	107 (8.3)	63 (9.0)	44 (7.6)
PR, N (%)			
Negative	390 (30.4)	232 (33.0)	158 (27.1)
Positive	841 (65.4)	447 (63.6)	394 (67.7)
Unknown	54 (4.2)	24 (3.4)	30 (5.2)
ER, N (%)			
Negative	294 (22.9)	159 (22.6)	135 (23.2)
Positive	934 (72.7)	520 (74.0)	414 (71.1)
Unknown	57 (4.4)	24 (3.4)	33 (5.7)
Radiation therapy, N (%)	802 (62.4)	355 (50.5)	447 (76.8)
Surgery, N (%)	1178 (91.7)	638 (90.8)	540 (92.8)
**Anti-cancer drug, N (%)**			
Targeted therapy	87 (6.8)	67 (9.5)	20 (3.4)
Anti-HER2	70 (5.4)	55 (7.8)	15 (2.6)
Kinase inhibitors	17 (1.4)	12 (1.7)	5 (1.8)
Chemotherapy	1258 (97.9)	684 (97.3)	574 (98.6)
Antimetabolites	308 (24.0)	175 (24.9)	133 (22.9)
Alkylating agents	1101 (85.7)	587 (83.5)	514 (88.3)
Alkaloids	14 (1.1)	6 (0.9)	8 (1.4)
Anthracyclines	902 (70.2)	449 (63.9)	453 (77.8)
Taxanes	155 (12.1)	95 (13.5)	60 (10.3)
**Comorbidity, N (%)**			
Pre-existing cardiac disease∗	241 (18.8)	147 (20.9)	94 (16.2)
Hypertension	290 (22.6)	174 (24.8)	116 (19.9)
Hyperlipidemia	230 (17.9)	144 (20.5)	86 (14.8)
Renal diseases	34 (2.6)	20 (2.8)	14 (2.4)
Chronic pulmonary diseases	98 (7.6)	74 (10.5)	24 (4.1)
Diabetes	183 (14.2)	99 (14.1)	84 (14.4)
Cerebrovascular disease	79 (6.1)	47 (6.7)	32 (5.5)
Liver diseases	150 (11.7)	90 (12.8)	60 (10.3)
Peripheral vascular disease	4 (0.3)	3 (0.4)	1 (0.2)
Dementia	31 (2.4)	22 (3.1)	9 (1.5)
Rheumatic disease	31 (2.4)	23 (3.3)	8 (1.4)
Peptic ulcer disease	69 (5.4)	47 (6.7)	22 (3.8)
**Concurrent medication (ATC code), N (%)**			
Biguanides (A10BA)	104 (8.1)	61 (8.7)	43 (7.4)
Statins (C10AA)	150 (11.7)	90 (12.8)	60 (10.3)
Antiplatelets (B01AC)	114 (8.9)	69 (9.8)	45 (7.7)
Beta blockers (C07AB)	84 (6.5)	53 (7.5)	31 (5.3)
Calcium channel blockers (C08CA)	121 (9.4)	73 (10.4)	48 (8.2)
Angiotensin II receptor blockers (C09CA)	119 (9.3)	77 (11.0)	42 (7.2)
Benzodiazepines (N05BA)	192 (14.9)	98 (13.9)	94 (16.2)
Sulfonylureas (A10BB)	49 (3.8)	16 (2.3)	33 (5.7)
DPP-4 inhibitors (A10BH)	44 (3.4)	27 (3.8)	17 (2.9)
Coxibs (M01AH)	76 (5.9)	43 (6.1)	33 (5.7)
**Laboratory test**			
Creatinine, Mean (SD)	0.8 (0.7)	0.7 (0.4)	0.8 (0.9)
BUN, Mean (SD)	15.5 (10.6)	15.1 (9.29)	17.6 (15.5)
Bilirubin, Mean (SD)	0.65 (0.54)	0.57 (0.21)	0.83 (0.89)
AST, Mean (SD)	25.1 (36.7)	25.1 (36.7)	23.0 (NA)
ALT, Mean (SD)	24.3 (26.5)	24.4 (26.5)	15.0 (3.61)
Cholesterol, Mean (SD)	200 (38.1)	232 (NA)	199 (38.2)
HDL, Mean (SD)	56.5 (16.1)	57.9 (17.2)	53.3 (12.9)
LDL, Mean (SD)	111 (31.9)	110 (32.6)	112 (30.9)
WBC, Mean (SD)	6.57 (2.44)	6.77 (2.47)	6.35 (2.38)
RBC, Mean (SD)	4.24 (0.513)	4.29 (0.522)	4.19 (0.497)
PLT, Mean (SD)	274 (92.0)	250 (75.1)	301 (101)
HCT, Mean (SD)	37.1 (4.17)	37.5 (4.46)	36.7 (3.76)
MCV, Mean (SD)	87.8 (7.28)	87.6 (7.67)	88.0 (6.80)
MCHC, Mean (SD)	33.7 (0.948)	33.6 (0.971)	33.8 (0.904)
MCH, Mean (SD)	30.1 (3.08)	30.4 (3.31)	29.8 (2.76)
Troponin I, Mean (SD)	0.09 (0.36)	0.03 (0.07)	0.18 (0.55)
BNP, Mean (SD)	266 (621)	845 (1130)	84.7 (161)
NT-pro BNP, Mean (SD)	1240 (1390)	1640 (1400)	50.0 (NA)
LVEF, Mean (SD)	70.0 (29.9)	67.8 (8.08)	72.2 (41.5)

**Note:** SD, Standard deviation; yrs., Years; IQR, Interquartile Range; BMI, Body mass index.

∗Pre-existing cardiac disease includes myocardial infarction, heart failure, arrhythmia, coronary artery disease, conduction disorder, and stroke diagnosed before the index date; DPP-4: Dipeptidyl peptidase 4; BUN, Blood urea nitrogen; AST, Aspartate aminotransferase; ALT, Alanine aminotransferase; HDL, High-density lipoprotein; LDL, Low-density lipoprotein; WBC, White blood count; RBC, Red blood count; PLT, Platelet; HCT, hematocrit; MCV, Mean corpuscular volume; MCHC, Mean corpuscular hemoglobin concentration; MCH, Mean corpuscular hemoglobin; BNP, B-type natriuretic peptide; NT-pro BNP, N-terminal pro b-type natriuretic peptide; LVEF: Left ventricular ejection fraction.

a The training set included the data from Taipei Medical University and Wan-Fang hospitals.

b The testing set included the data from Shuang Ho hospital.

### 3.2 Model performance

**Fig. 2:**
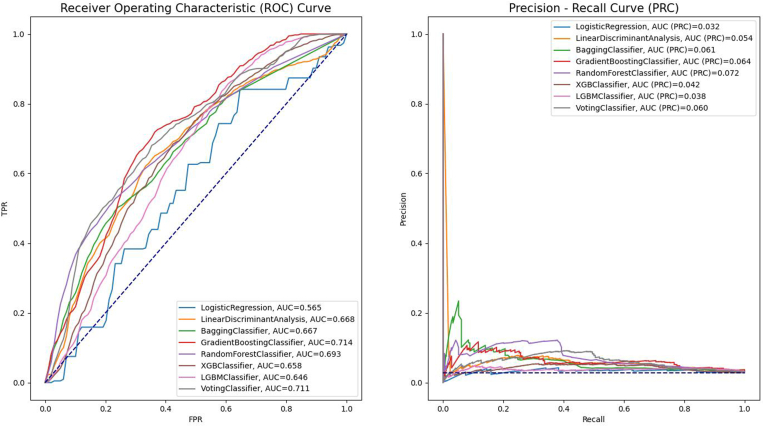
Performance of the prediction models in the testing dataset.

The receiver operator characteristic curves of various models are demonstrated in Fig. 2. LR was observed with the lowest AUC (0.57). The other machine learning models exhibited moderate performance (AUC ranged from 0.65 to 0.71). Models with the highest AUC were Gradient Boosting and Voting Classifier (0.71). Regarding other metrics, Voting Classifier had an overall better performance compared to Gradient Boosting (i.e., accuracy: 0.84 versus 0.64; precision 0.09 versus 0.06; recall: 0.49 versus 0.75; and F1-score: 0.15 versus 0.11). The performance of other models is shown in Table 2. Most models, except for LR and LGBM, had AUPRCs at least 2 times higher than the baseline of random classifier.

**Table 2:** Comparison of predictive performance of different models.

**Model**	**AUC**	**Accuracy**	**Sensitivity**	**Specificity**	**NPV**	**PPV**	**F1-score**
Logistic Regression	0.57	0.37	0.84	0.36	0.99	0.04	0.07
Linear Discriminant Analysis	0.67	0.64	0.69	0.64	0.99	0.05	0.1
Bagging Classifier	0.67	0.44	0.76	0.43	0.98	0.04	0.07
Gradient Boosting Classifier	0.71	0.64	0.75	0.63	0.99	0.06	0.11
Random Forest Classifier	0.69	0.62	0.69	0.62	0.99	0.05	0.09
XGB Classifier	0.66	0.64	0.66	0.63	0.98	0.05	0.09
LGBM Classifier	0.65	0.42	0.86	0.41	0.99	0.04	0.08
Voting Classifier	0.71	0.84	0.49	0.85	0.98	0.09	0.15

**Note:** AUC, area under the curve; LGBM, Light Gradient Boosting Machine; XGB, Extreme Gradient Boosting; NPV, negative prediction value; PPV, positive prediction value.

Voting Classifier was selected as the optimal classifier for further experiment. The model was applied to patients with a specific type of anti-cancer drug. 6 classes of drug were antimetabolites, alkylating agents, alkaloids, anthracyclines, taxanes, and anti-HER2 therapies (Fig. S1). Model performance for each type of therapy was relatively good, especially for alkylating agents and anti-HER2 therapy (AUC: 0.79 and 0.76 respectively). Average precision of the model for alkylating agents was 10 times higher than the baseline (0.245 vs 0.024).

### 3.3 Importance score

**Fig. 3:**
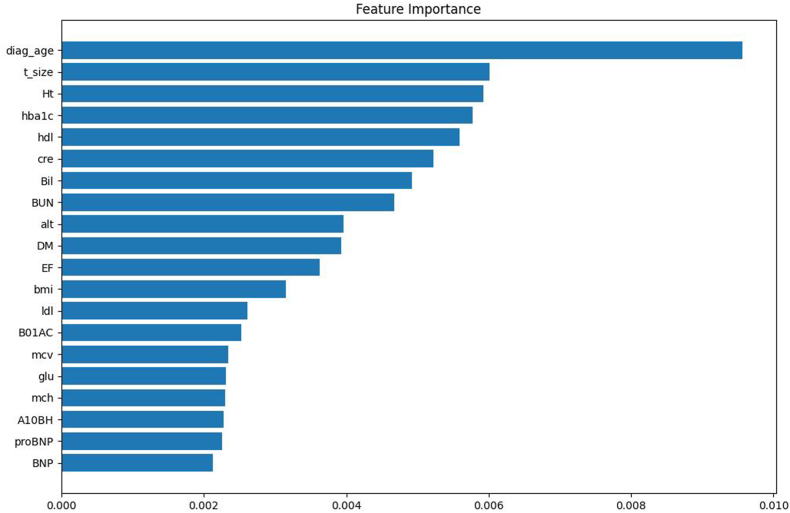
Top 20 important features in the Voting Classifier model. **Note:** diag_age: age; t_size: tumor size; Ht: hypertension; hba1c: HbA1c; hdl: high-density lipoprotein; cre: creatinine; Bil: bilirubin; BUN: blood urea nitrogen; alt: alanine aminotransferase; DM: diabetes; EF: left ventricular ejection fraction; bmi: body mass index; ldl: low-density lipoprotein; B01AC: antiplatelets; mcv: mean corpuscular volume; glu: glucose; mch: mean corpuscular hemoglobin; A10BH: DPP-4 inhibitors; proBNP: NT-pro BNP; BNP: b-type natriuretic peptide.

Importance score was graphed to determine the association between features and cardiac outcome in the best model (Fig. 3). Ten features with the highest important scores were age, tumor size, hypertension, HbA1c, HDL, creatinine, bilirubin, BUN, ALT, and diabetes.

## 4 Discussion

In this study, machine learning algorithms were applied to predict cardiovascular events following systemic therapy among breast cancer population. Most models showed relatively good performance. Overall, Voting Classifier was the best model (AUC: 0.71, accuracy: 0.84; precision: 0.09, recall: 0.49, F1-score: 0.15). Another analysis was made to investigate how well the model perform for specific anti-cancer drug. In addition, contribution of each feature to the prediction model was also examined.

To date, several risk scores have been proposed to assess cardiovascular risk for breast cancer population. Most studies focused on trastuzumab or anthracycline users and provided a scoring tool to generally evaluate the cardiovascular risk they might get. Age and comorbidities were the most often used predictors [[23], [24], [25], [26], [27]]. One study included regional or distant invasion as predictors [27]. Some studies considered dose of anticancer drugs [24,27] and cardiac function parameter such as baseline LVEF [23,26,28], In these studies, a risk score was assigned to each risk factor. The prediction models were constructed of a few numbers of risk factors using logistic regression [23,[28], [29], [30], [31]] or Cox regression [24,32] as the main algorithm. Discrimination ability was quantified by C-index or AUROC. In general, the models had moderate discrimination (C-index: 0.70–0.79) [23,26,27]. The highest C-index was observed with CHEMO-RADIAT score (C-index = 0.87) [24]. Feng et al. found that predictive model using deceleration capacity of heart rate had a better discrimination than model using baseline LVEF (AUROC: 0.88 vs 0.77, respectively) [31]. Other models had low AUROC, ranging from 0.56 to 0.70 [29,30,33].

Prediction model for cardiotoxicity in breast cancer patients using artificial intelligence approach is limited. Chang et al. reported machine learning models to predict cardiac dysfunction induced by anthracycline [34]. Among the investigated models, Multilayer Perceptron showed an AUC of 0.66 for cardiac dysfunction outcome and an AUC of 0.79 for heart failure with reduced ejection fraction outcome. However, as the data was not stratified during sampling, the model performance might be overestimated. In our study, stratifying by outcome ensured that each class is proportionately presented in both the training and testing sets. This approach prevents any outcome class from being overrepresented or underrepresented, which could lead to biased model training and unreliable performance estimates. Furthermore, unlike previous studies, we took into consideration various cardiac outcomes beyond heart dysfunction. Heart failure is a late toxicity which may be a result of less severe conditions such as arrhythmia, conduction disorder, coronary artery disease. In studies that focused on cardiac dysfunction, the follow up duration was up to 3 or 7 years [[24], [25], [26], [27]]. In the current study, we focused on cardiac events that happened within 1 year since drug use. Detecting early onset cardiovascular events may help to prevent life-threatening conditions. In addition, previous studies only focused on either trastuzumab or anthracycline. Since there is evidence for cardiotoxicity of other anticancer drugs including antimetabolites, alkylating agents, taxanes [[35], [36], [37]]; we also explored our model for these medications.

Our model helps to differentiate between patients who are at increased risk of having cardiotoxicity and patients who are at low risk. According to ESC guidelines, echocardiography should be performed every three months in patients receiving trastuzumab, even in the low-risk group [38]. On the other hand, some studies suggested that intensive cardiac monitoring might not be essential for patients who deemed to be at low risk [[39], [40], [41]]. Our model facilitates a more targeted screening approach, where women identified as high-risk can undergo more frequent screenings and women identified as low risk can undergo less frequent screenings. Moreover, this model was built on patient's time series data, enabling it to assess not only new anticancer drug users but also patients who have been using the medication over an extended period. Patients' conditions change over time, especially cancer characteristics and laboratory indexes. Before every cycle of chemotherapy, physicians can use the tool to evaluate cardiovascular risk and enable simultaneous surveillance for individual patients.

This study supports the conclusions drawn from previous research. Age, hypertension, and diabetes are well-known risk factors for cardiovascular disease in the general population as well as in patients receiving anticancer therapy [[42], [43], [44], [45]]. These variables were also identified as important features of the current model. Another strong predictor was tumor size. Large tumor increased the risk of CVD death in breast cancer patients receiving chemotherapy [46]. Routine blood test values, such as HbA1c, HDL, bilirubin, creatinine, BUN and ALT, significantly contributed to the predictive power of this model. HbA1c is not only a diagnostic index for type 2 diabetes but also a predictor for cardiovascular disease in both diabetes and non-diabetes populations. A cohort study of 608,474 individuals without diabetes at baseline showed that elevated HbA1c was associated with an increased risk of CVD outcomes [47]. In other studies, a J-shaped relationship was found between HbA1c levels and heart failure incidence [48,49]. High HDL level was associated with a low anthracycline-induced cardiotoxicity risk in patients with diffuse large B-cell lymphoma [50]. In addition, HDL showed a protective effect against doxorubin-induced cardiotoxicity via scavenger receptor class B type 1, phosphatidylinositol 3-kinase, and Akt-dependent manner [51]. According to recent meta-analysis and population-based studies [[52], [53], [54]], serum bilirubin is inversely associated with CVDs risk in general population. Associations between creatinine, BUN, ALT and cardiovascular disease were also reported [[55], [56], [57], [58]]. Findings from this study offer new insights for CVD risk management in breast cancer patients undergoing chemotherapy, as the importance of routine lab test is often overlooked.

There are several limitations in this study. Firstly, the study utilized a secondary dataset which was previously employed to develop a prediction model for mortality in breast cancer patients [59]. Many laboratory features had a high proportion of missing data. MICE imputation was applied to handle this issue. However, future study with a prospective design could provide high-quality input, potentially enhancing model performance. Secondly, small sample size is another factor that affects the model's performance, especially when cardiovascular outcome is rare. This limitation can be overcome in the future as we expand this study to other cancer populations. Thirdly, although external validation was conducted, the data was also from a hospital located in northern Taiwan. To ensure generalizability of the results, the model should be validated by data from other areas or other countries. Fourthly, although SHAP importance provides an overview about the relationship between variables and outcome, it does not reveal direction of the association between them. Deeper investigation into how each factor is associated with the outcome is necessary. Fifthly, the inclusion of additional risk factors, such as endocrine therapy and laterality, did not improve the model's performance (Fig. S2, Table S4). Therefore, these variables were not included in the final model. One possible reason is that these two variables may not be highly relevant for prediction. Additionally, the imbalanced data (with an outcome ratio of 0.024) makes achieving high performance challenging. Lastly, since this study included patients with more than one type of anticancer drug, we could not examine the impact of the dose on cardiac outcomes. Further study including dose as a predictor for each class of drug is needed.

## 5 Conclusion

In the current study, we developed various machine learning models to predict cardiovascular risk after treatment with chemotherapy and targeted agents in breast cancer patients. Among the models, Voting Classifier showed the best performance. As the model was built on a time-series concept, it could support clinicians to assess cardiovascular risk for individual patients before, during, or after receiving anticancer drug. Additional research investigating the practical implementation of this model in clinical settings is necessary.

## CRediT authorship contribution statement

**Quynh T.N. Nguyen:** Writing – original draft, Software, Methodology, Investigation, Formal analysis, Data curation, Conceptualization. **Shwu-Jiuan Lin:** Writing – review & editing, Supervision, Methodology. **Phung-Anh Nguyen:** Writing – review & editing, Methodology, Investigation, Formal analysis, Conceptualization. **Phan Thanh Phuc:** Writing – review & editing, Software.**Min-Huei Hsu:** Writing – review & editing, Methodology, Resources, Project administration, Funding acquisition. **Chun-Yao Huang:** Writing – review & editing, Methodology. **Chin-Sheng Hung:** Writing – review & editing, Methodology. **Christine Y. Lu:** Writing – review & editing, Methodology. **Jason C. Hsu:** Writing – review & editing, Supervision, Resources, Project administration, Methodology, Investigation, Funding acquisition, Conceptualization.

## Ethical approval

This study was approved by the Taipei Medical University–Joint Institutional Review Board (IRB No. N202201089). All data were de-identified before the analysis and thus informed consent was not required.

## Funding sources

This work was supported by 10.13039/100020595Taiwan National Science and Technology Council (grant no. NSTC 113-2321-B-038-006).

## Declaration of competing interest

All authors declare no conflict of interest.

## Glossary

ALT: Alanine aminotransferase
AUPRC: Area under the precision-recall curve
AUROC: Area under the receiver operating characteristic curve
BMI: Body mass index
BUN: Blood urea nitrogen
CAD: Coronary artery disease
CVD: Cardiovascular disease
DPP-4: Dipeptidyl peptidase 4
ER: Estrogen receptor
ESC: European society of cardiology
GBM: Gradient boosting machine
HDL: High-density lipoprotein
HER2: Human epidermal growth factor receptor 2
HF: Heart failure
LDA: Linear discriminant analysis
LGBM: Light gradient boosting machine
LR: Logistic regression
LVEF: Left ventricular ejection fraction
MI: Myocardial infarction
MICE: Multivariate imputation by chained-equations
NPV: Negative predictive value
PPV: Positive predictive value
PR: Progesterone receptor
RF: Random Forest
SHAP: Shapley additive explanations
TCR: Taiwan cancer registry
TMUCRD: Taipei Medical University clinical research database
