# Supplementary material for: Early prediction of cardiovascular events following treatments in female breast cancer patients: Application of real-world data and artificial intelligence
Source: Breast. 2025 Mar 10;81:104438. doi: 10.1016/j.breast.2025.104438 (PMC11992427; doi:10.1016/j.breast.2025.104438)
Supplement: Multimedia component 1 [file mmc1.docx]

**Table S1. Detailed baseline characteristics of the Study Population**

|  | **Overall  (n=1285)** | **Training cohort^a^  (n=703)** | **Testing cohort^b^  (n=582)** |
| --- | --- | --- | --- |
| **Cardiac outcomes, N (%)** | 36 (2.8) | 22 (3.1) | 14 (2.4) |
| Arrhythmia | 12 (0.9) | 9 (1.3) | 3 (0.5) |
| CAD | 14 (1.1) | 10 (1.4) | 4 (0.7) |
| HF | 9 (0.7) | 4 (0.6) | 5 (0.9) |
| Conduction disorder | 1 (0.1) | 1 (0.1) | 0 (0) |
| Myocardial infarction | 0 (0) | 0 (0) | 0 (0.0) |
| Stroke | 7 (0.5) | 3 (0.4) | 4 (0.7) |
| Newly diagnosed cardiac events | 18 (1.4) | 8 (1.1) | 10 (1.7) |
| **Demographic information** |  |  |  |
| Age, Mean (SD), yrs. | 56.1 (11.5) | 56.6 (12.1) | 55.5 (10.7) |
| BMI, Mean (SD), kg/m^2^ | 24.3 (4.0) | 24.1 (4.0) | 24.6 (4.1) |
| Smoking, N (%) |  |  |  |
| No | 1129 (87.9) | 627 (89.2) | 502 (86.3) |
| Yes | 94 (7.3) | 46 (6.5) | 48 (8.2) |
| Unknown | 62 (4.8) | 30 (4.3) | 32 (5.5) |
| Drinking, N (%) |  |  |  |
| No | 1137 (88.5) | 625 (88.9) | 512 (88.0) |
| Yes | 69 (5.4) | 45 (6.4) | 24 (4.1) |
| Unknown | 79 (6.1) | 33 (4.7) | 46 (7.9) |
| **Cancer condition** |  |  |  |
| Tumor size, mm |  |  |  |
| Mean (SD) | 29.3 (20.0) | 29.6 (21.1) | 28.9 (18.4) |
| Median [IQR] | 24.0 [16.0, 35.0] | 24.0 [16.0, 35.0] | 24.0 [17.0, 35.0] |
| Missing | 34 (2.6) | 16 (2.3) | 18 (3.1) |
| Cancer stage, N (%) |  |  |  |
| stage = 0 | 47 (3.7) | 29 (4.1) | 18 (3.1) |
| stage = 1 | 357 (27.8) | 214 (30.4) | 143 (24.6) |
| stage = 2 | 636 (49.5) | 355 (50.5) | 281 (48.3) |
| stage = 3 | 78 (6.1) | 46 (6.5) | 32 (5.5) |
| stage = 4 | 96 (7.5) | 50 (7.1) | 46 (7.9) |
| Unknown | 71 (5.5) | 9 (1.3) | 62 (10.7) |
| HER2, N (%) |  |  |  |
| Negative | 820 (63.8) | 447 (63.6) | 373 (64.1) |
| Positive | 358 (27.9) | 193 (27.5) | 165 (28.4) |
| Unknown | 107 (8.3) | 63 (9.0) | 44 (7.6) |
| PR, N (%) |  |  |  |
| Negative | 390 (30.4) | 232 (33.0) | 158 (27.1) |
| Positive | 841 (65.4) | 447 (63.6) | 394 (67.7) |
| Unknown | 54 (4.2) | 24 (3.4) | 30 (5.2) |
| ER, N (%) |  |  |  |
| Negative | 294 (22.9) | 159 (22.6) | 135 (23.2) |
| Positive | 934 (72.7) | 520 (74.0) | 414 (71.1) |
| Unknown | 57 (4.4) | 24 (3.4) | 33 (5.7) |
| Laterality, N (%) |  |  |  |
| Right | 639 (49.7) | 348 (49.5) | 291 (50.0) |
| Left | 601 (46.8) | 334 (47.5) | 267 (45.9) |
| Bilateral | 28 (2.2) | 18 (2.6) | 10 (1.7) |
| Unknown | 17 (1.3) | 3 (0.4) | 14 (2.4) |
| Radiation therapy, N (%) |  |  |  |
| No | 459 (35.7) | 333 (47.4) | 126 (21.6) |
| Yes | 802 (62.4) | 355 (50.5) | 447 (76.8) |
| Unknown | 24 (1.9) | 15 (2.1) | 9 (1.5) |
| Surgery, N (%) |  |  |  |
| No | 90 (7.0) | 56 (8.0) | 34 (5.8) |
| Yes | 1178 (91.7) | 638 (90.8) | 540 (92.8) |
| Unknown | 17 (1.3) | 9 (1.3) | 8 (1.4) |
| **Anti-cancer drug, N (%)** |  |  |  |
| Targeted therapy | 87 (6.8) | 67 (9.5) | 20 (3.4) |
| Anti-HER2 | 70 (5.4) | 55 (7.8) | 15 (2.6) |
| Kinase inhibitors | 17 (1.4) | 12 (1.7) | 5 (1.8) |
| Chemotherapy | 1258 (97.9) | 684 (97.3) | 574 (98.6) |
| Antimetabolites | 308 (24.0) | 175 (24.9) | 133 (22.9) |
| Alkylating agents | 1101 (85.7) | 587 (83.5) | 514 (88.3) |
| Alkaloids | 14 (1.1) | 6 (0.9) | 8 (1.4) |
| Anthracyclines | 902 (70.2) | 449 (63.9) | 453 (77.8) |
| Taxanes | 155 (12.1) | 95 (13.5) | 60 (10.3) |
| Endocrine therapy | 14 (1.1) | 7 (1.1) | 7 (1.2) |
| **Comorbidity, N (%)** |  |  |  |
| Pre-existing cardiac disease^*^ | 241 (18.8) | 147 (20.9) | 94 (16.2) |
| Hypertension | 290 (22.6) | 174 (24.8) | 116 (19.9) |
| Hyperlipidemia | 230 (17.9) | 144 (20.5) | 86 (14.8) |
| Renal diseases | 34 (2.6) | 20 (2.8) | 14 (2.4) |
| Chronic pulmonary diseases | 98 (7.6) | 74 (10.5) | 24 (4.1) |
| Diabetes | 183 (14.2) | 99 (14.1) | 84 (14.4) |
| Cerebrovascular disease | 79 (6.1) | 47 (6.7) | 32 (5.5) |
| Liver diseases | 150 (11.7) | 90 (12.8) | 60 (10.3) |
| Peripheral vascular disease | 4 (0.3) | 3 (0.4) | 1 (0.2) |
| Dementia | 31 (2.4) | 22 (3.1) | 9 (1.5) |
| Rheumatic disease | 31 (2.4) | 23 (3.3) | 8 (1.4) |
| Peptic ulcer disease | 69 (5.4) | 47 (6.7) | 22 (3.8) |
| **Concurrent medication (ATC code), N (%)** |  |  |  |
| Biguanides (A10BA) | 104 (8.1) | 61 (8.7) | 43 (7.4) |
| Statins (C10AA) | 150 (11.7) | 90 (12.8) | 60 (10.3) |
| Antiplatelets (B01AC) | 114 (8.9) | 69 (9.8) | 45 (7.7) |
| Beta blockers (C07AB) | 84 (6.5) | 53 (7.5) | 31 (5.3) |
| Calcium channel blockers (C08CA) | 121 (9.4) | 73 (10.4) | 48 (8.2) |
| Angiotensin II receptor blockers (C09CA) | 119 (9.3) | 77 (11.0) | 42 (7.2) |
| Benzodiazepines (N05BA) | 192 (14.9) | 98 (13.9) | 94 (16.2) |
| Sulfonylureas (A10BB) | 49 (3.8) | 16 (2.3) | 33 (5.7) |
| DPP-4 inhibitors (A10BH) | 44 (3.4) | 27 (3.8) | 17 (2.9) |
| Coxibs (M01AH) | 76 (5.9) | 43 (6.1) | 33 (5.7) |
| **Laboratory test** |  |  |  |
| Creatinine, Mean (SD)  Missing, N (%) | 0.8 (0.7)  626 (48.7) | 0.7 (0.4)  426 (60.6) | 0.8 (0.9)  200 (34.4) |
| BUN, Mean (SD)  Missing, N (%) | 15.5 (10.6)  794 (61.8) | 15.1 (9.3)  297 (42.2) | 17.6 (15.5)  497 (85.4) |
| Bilirubin, Mean (SD)  Missing, N (%) | 0.65 (0.54)  1055 (82.1%) | 0.57 (0.21)  544 (77.4%) | 0.83 (0.89)  511 (87.8%) |
| AST, Mean (SD)  Missing, N (%) | 25.1 (36.7)  966 (75.2%) | 25.1 (36.7)  385 (54.8%) | 23.0 (NA)  581 (99.8%) |
| ALT, Mean (SD)  Missing, N (%) | 24.3 (26.5)  691 (53.8%) | 24.4 (26.5)  112 (15.9%) | 15.0 (3.61)  579 (99.5%) |
| Cholesterol, Mean (SD)  Missing, N (%) | 200 (38.1)  1153 (89.7%) | 232 (NA)  702 (99.9%) | 199 (38.2)  451 (77.5%) |
| HDL, Mean (SD)  Missing, N (%) | 56.5 (16.1)  1122 (87.3%) | 57.9 (17.2)  591 (84.1%) | 53.3 (12.9)  531 (91.2%) |
| LDL, Mean (SD)  Missing, N (%) | 111 (31.9)  1023 (79.6%) | 110 (32.6)  549 (78.1%) | 112 (30.9)  474 (81.4%) |
| WBC, mean (SD)  Missing, N (%) | 6.57 (2.44)  180 (14.0%) | 6.77 (2.47)  114 (16.2%) | 6.35 (2.38)  66 (11.3%) |
| RBC, mean (SD)  Missing, N (%) | 4.24 (0.513)  182 (14.2%) | 4.29 (0.522)  115 (16.4%) | 4.19 (0.497)  67 (11.5%) |
| PLT, mean (SD)  Missing, N (%) | 274 (92.0)  182 (14.2%) | 250 (75.1)  116 (16.5%) | 301 (101)  66 (11.3%) |
| HCT, mean (SD)  Missing, N (%) | 37.1 (4.17)  182 (14.2%) | 37.5 (4.46)  115 (16.4%) | 36.7 (3.76)  67 (11.5%) |
| MCV, mean (SD)  Missing, N (%) | 87.8 (7.28)  182 (14.2%) | 87.6 (7.67)  115 (16.4%) | 88.0 (6.80)  67 (11.5%) |
| MCHC, mean (SD)  Missing, N (%) | 33.7 (0.948)  183 (14.2%) | 33.6 (0.971)  116 (16.5%) | 33.8 (0.904)  67 (11.5%) |
| MCH, mean (SD)  Missing, N (%) | 30.1 (3.08)  183 (14.2%) | 30.4 (3.31)  116 (16.5%) | 29.8 (2.76)  67 (11.5%) |
| Troponin I, Mean (SD)  Missing N (%) | 0.09 (0.36)  1248 (97.1%) | 0.03 (0.07)  682 (97.0%) | 0.18 (0.55)  566 (97.3%) |
| BNP, Mean (SD)  Missing, N (%) | 266 (621)  1264 (98.4%) | 845 (1130)  698 (99.3%) | 84.7 (161)  566 (97.3%) |
| NT-pro BNP, Mean (SD)  Missing N (%) | 1240 (1390)  1281 (99.7%) | 1640 (1400)  700 (99.6%) | 50.0 (NA)  581 (99.8%) |
| LVEF, Mean (SD)  Missing, N (%) | 70.0 (29.9)  475 (37.0) | 67.8 (8.08)  298 (42.4) | 72.2 (41.5)  177 (30.4) |

**Note:** SD, Standard deviation; yrs., Years; IQR, Interquartile Range; BMI, Body mass index; ^*^ Pre-existing cardiac disease includes myocardial infarction, heart failure, arrhythmia, coronary artery disease, conduction disorder, and stroke diagnosed before the index date ; BUN, Blood urea nitrogen; AST, Aspartate aminotransferase; ALT, Alanine aminotransferase; HDL, High-density lipoprotein; LDL, Low-density lipoprotein; WBC, White blood count; RBC, Red blood count; PLT, Platelet; HCT, hematocrit; MCV, Mean Corpuscular Volume; MCHC, Mean corpuscular hemoglobin concentration; MCH, Mean corpuscular hemoglobin; BNP, B-type natriuretic peptide; NT-pro BNP, N-terminal pro b-type natriuretic peptide; LVEF: Left ventricular ejection fraction; ^a^The training set included the data from Taipei Medical University and Wan-Fang hospitals; ^b^The testing set included the data from Shuang Ho hospital.

**Table S2. The associations between different features and the outcome at the patient baseline**

|  | **crude OR (95%CI)** | **crude P value** | **adj. OR (95%CI)** | **P (Wald's test)** |
| --- | --- | --- | --- | --- |
| **Demographic** |  |  |  |  |
| Age^*^ | 1.07 (1.04 - 1.09) | < 0.001 | 1.00 (0.97 - 1.04) | 0.83 |
| BMI^*^ | 1.06 (1.01 - 1.11) | 0.012 | 0.96 (0.88 - 1.04) | 0.299 |
| Smoking (Yes vs. No) | 0.46 (0.16 - 1.31) | 0.146 | 0.53 (0.1 - 2.94) | 0.469 |
| Drinking (Yes vs. No) | 0.18 (0.02 - 1.35) | 0.096 | 0.27 (0.02 - 3.13) | 0.296 |
| **Cancer condition** |  |  |  |  |
| Tumor size^*^ | 1.00 (0.99 - 1.01) | 0.921 | 1.00 (0.98 - 1.02) | 0.891 |
| Cancer stage (Ref. = 0) |  |  |  |  |
| 1 | 0.83 (0.26 - 2.63) | 0.753 | 0.68 (0.11 - 4.2) | 0.676 |
| 2 | 0.76 (0.25 - 2.32) | 0.625 | 0.62 (0.1 - 3.84) | 0.605 |
| 3 | 0.62 (0.15 - 2.6) | 0.518 | 0.71 (0.06 - 8.42) | 0.787 |
| 4 | 0.77 (0.19 - 3.06) | 0.711 | 0.24 (0.01 - 4.13) | 0.326 |
| Unknown | 0.39 (0.08 - 1.9) | 0.241 | 0.44 (0.04 - 4.82) | 0.499 |
| HER2 (Positive vs. Negative) | 1.45 (0.91 - 2.3) | 0.119 | 1.27 (0.6 - 2.65) | 0.533 |
| PR (Positive vs. Negative) | 0.8 (0.51 - 1.26) | 0.343 | 1.55 (0.58 - 4.09) | 0.381 |
| ER (Positive vs. Negative) | 0.7 (0.43 - 1.13) | 0.141 | 0.34 (0.12 - 0.91) | 0.032 |
| **Cancer treatment (Yes vs. No)** |  |  |  |  |
| Radiation therapy | 0.65 (0.42 - 1.01) | 0.055 | 0.79 (0.39 - 1.61) | 0.518 |
| Surgery | 0.78 (0.32 - 1.93) | 0.595 | 1.18 (0.13 - 10.33) | 0.882 |
| Chemotherapy | 1.11 (0.14 - 9.14) | 0.921 | 22.76 (0.98 - 531.31) | 0.052 |
| Targeted therapy | 1.16 (0.47 - 2.84) | 0.752 | 1.78 (0.37 - 8.65) | 0.475 |
| **Comorbidities (Yes vs. No)** |  |  |  |  |
| Pre-existing cardiac disease | 23.56 (13.49 - 41.15) | < 0.001 | 16.88 (7.56 - 37.69) | < 0.001 |
| Hypertension | 7.84 (4.91 - 12.52) | < 0.001 | 1.79 (0.77 - 4.13) | 0.174 |
| Hyperlipidemia | 4.55 (2.9 - 7.14) | < 0.001 | 0.72 (0.32 - 1.6) | 0.416 |
| Renal disease | 4.14 (1.67 - 10.27) | 0.002 | 2.57 (0.49 - 13.52) | 0.265 |
| Chronic pulmonary disease | 2.36 (1.26 - 4.44) | 0.008 | 0.66 (0.25 - 1.74) | 0.399 |
| Diabetes | 3.88 (2.4 - 6.26) | < 0.001 | 1.46 (0.5 - 4.28) | 0.489 |
| Cerebrovascular disease | 8.17 (4.45 - 14.98) | < 0.001 | 0.79 (0.27 - 2.3) | 0.666 |
| Liver disease | 1.7 (0.99 - 2.94) | 0.056 | 0.73 (0.31 - 1.7) | 0.465 |
| Dementia | 23.53 (7.5 - 73.87) | < 0.001 | 2.79 (0.47 - 16.54) | 0.258 |
| Rheumatic disease | 2.02 (0.72 - 5.66) | 0.179 | 0.67 (0.13 - 3.49) | 0.638 |
| Peptic ulcer disease | 1.56 (0.66 - 3.67) | 0.307 | 1.05 (0.33 - 3.41) | 0.931 |
| **Medications (Yes vs. No)** |  |  |  |  |
| Biguanides (A10BA) | 3.48 (1.97 - 6.17) | < 0.001 | 0.71 (0.17 - 2.99) | 0.646 |
| Statins (C10AA) | 8.06 (4.99 - 13.01) | < 0.001 | 1.31 (0.56 - 3.04) | 0.536 |
| Antiplatelets (B01AC) | 15.45 (9.1 - 26.23) | < 0.001 | 4.29 (1.87 - 9.87) | < 0.001 |
| Beta blockers (C07AB) | 5.39 (3.09 - 9.39) | < 0.001 | 1.28 (0.55 - 2.98) | 0.56 |
| Calcium channel blockers (C08CA) | 4.44 (2.61 - 7.56) | < 0.001 | 1.2 (0.48 - 2.96) | 0.698 |
| Angiotensin II receptor blockers (C09CA) | 6.66 (4 - 11.08) | < 0.001 | 2.16 (0.87 - 5.33) | 0.095 |
| Benzodiazepines (N05BA) | 3.44 (2.12 - 5.57) | < 0.001 | 2.32 (1.11 - 4.84) | 0.025 |
| Sulfonylureas (A10BB) | 1.48 (0.59 - 3.71) | 0.397 | 0.45 (0.08 - 2.42) | 0.353 |
| DPP-4 inhibitors (A10BH) | 5.91 (2.81 - 12.43) | < 0.001 | 1.16 (0.28 - 4.71) | 0.841 |
| Coxibs (M01AH) | 1.74 (0.81 - 3.75) | 0.159 | 0.48 (0.15 - 1.5) | 0.206 |
| **Lab test^*^** |  |  |  |  |
| WBC | 1.00 (0.99 - 1.00) | 0.603 | 1.00 (1.00 - 1.01) | 0.027 |
| RBC | 0.99 (0.99 – 1.00) | 0.016 | 1.00 (0.97 - 1.03) | 0.925 |
| PLT | 0.99 (0.99 – 1.00) | 0.002 | 0.99 (0.99 - 1.00) | 0.074 |
| HCT | 0.99 (0.99 - 1.00) | 0.205 | 1.00 (0.97 - 1.04) | 0.973 |
| MCV | 1.01 (1.001 - 1.009) | 0.02 | 0.99 (0.98 - 1.01) | 0.573 |
| MCHC | 1.01 (0.98 - 1.03) | 0.553 | 1.02 (0.97 - 1.07) | 0.486 |
| MCH | 1.01 (1 - 1.02) | 0.006 | 1.01 (0.99 - 1.03) | 0.437 |
| LVEF | 1.003 (0.998 - 1.007) | 0.206 | 1.005 (0.998 - 1.011) | 0.157 |

**Note:** *continuous variable

**Table S3. International Classification of Diseases 9 and 10 for six cardiac outcomes**

| **Outcome** | **ICD-9** | **ICD-10** |
| --- | --- | --- |
| Arrhythmia | 427.x | I46.9, I47.1, I47.2, I47.9, I48.91, I48.92, I49.01, I49.02, I49.40, I49.49, I49.1, I49.3, I49.5, R00.1, I49.8, I49.9 |
| CAD | 414.x | I25.10, I25.3, I25.41, I25.42, I25.5, I25.810, I25.811, I25.812, I25.82, I25.83, I25.84, I25.89, I25.9 |
| Heart failure | 398.91, 402.01, 402.11, 402.91, 404.01, 404.03, 404.11, 404.13, 404.91, 404.93, 425.4 - 425.9, 428.x | I09.9, I11.0, I13.0, I13.2, I25.5, I42.0, I42.5 - I42.9, I43.x, I50.x, P29.0 |
| MI | 410.x, 412.x | I21.x, I22.x, I25.2 |
| Conduction disorder | 426.x | I44.2, I44.30, I44.0, I44.1, I44.30, I44.39, I44.4-5, I44.60, I44.69, I44.7, I45.10, I45.4, I45.2, I45.3, I45.5, I45.6, I45.81, I45.89, I45.9 |
| Stroke | 431-435, 437.01 | I61-I66, I67.28 |

**Table S4. Model performance with two additional features: laterality and endocrine therapy**

| **Model** | **AUC** | **Accuracy** | **Sensitivity** | **Specificity** | **NPV** | **PPV** | **F1-score** |
| --- | --- | --- | --- | --- | --- | --- | --- |
| Logistic Regression | 0.58 | 0.89 | 0.25 | 0.91 | 0.98 | 0.08 | 0.12 |
| Linear Discriminant Analysis | 0.62 | 0.6 | 0.68 | 0.6 | 0.98 | 0.05 | 0.09 |
| Bagging Classifier | 0.66 | 0.8 | 0.49 | 0.81 | 0.98 | 0.07 | 0.12 |
| Gradient Boosting Classifier | 0.67 | 0.61 | 0.68 | 0.61 | 0.98 | 0.05 | 0.09 |
| Random Forest Classifier | 0.68 | 0.75 | 0.52 | 0.76 | 0.98 | 0.06 | 0.11 |
| XGB Classifier | 0.69 | 0.48 | 0.79 | 0.47 | 0.99 | 0.04 | 0.08 |
| LGBM Classifier | 0.65 | 0.54 | 0.78 | 0.54 | 0.99 | 0.05 | 0.09 |
| Voting Classifier | 0.67 | 0.79 | 0.46 | 0.8 | 0.98 | 0.06 | 0.11 |

**
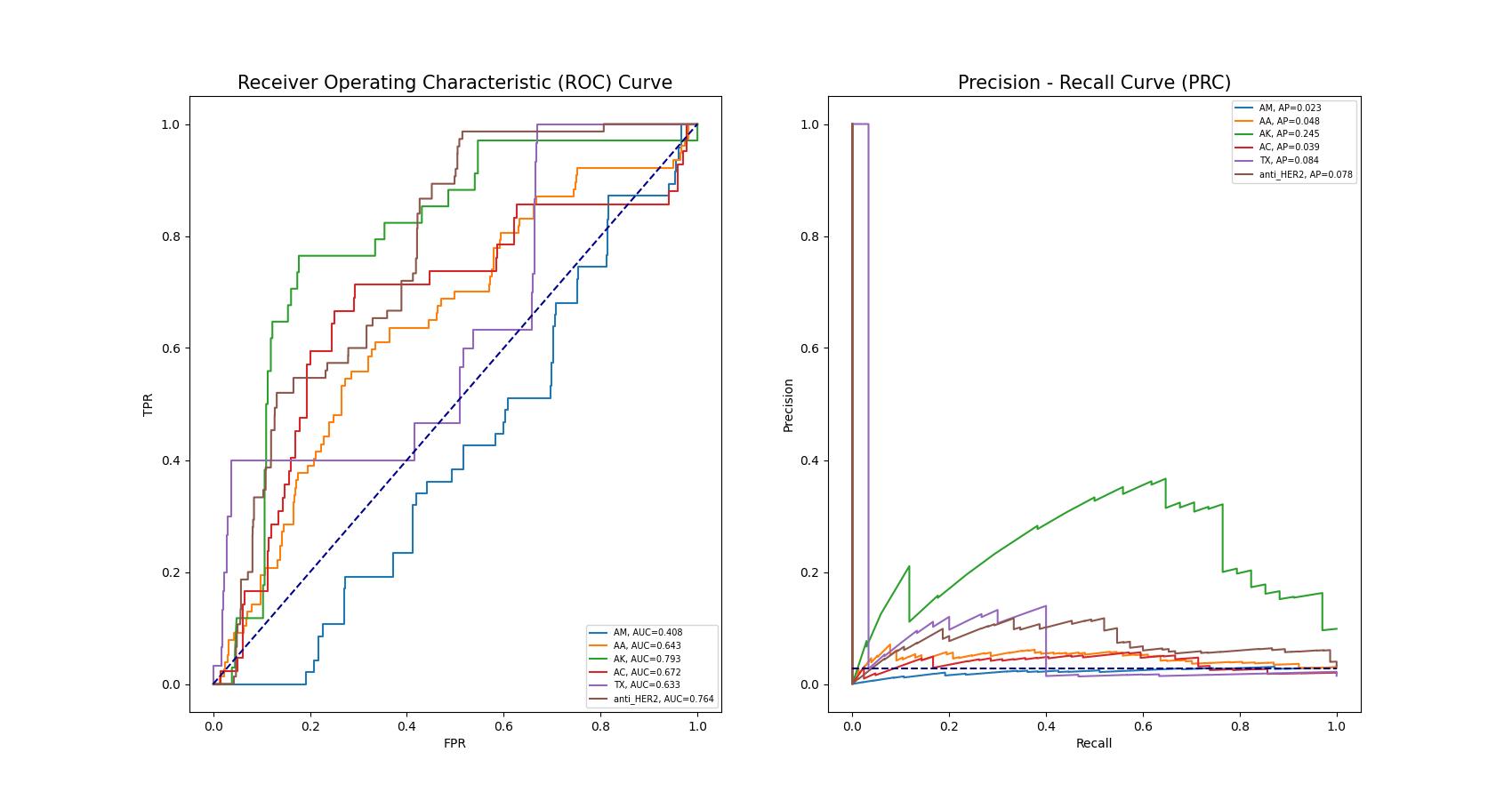
**

**Figure S1. Model performance for different anticancer drugs**

AM: antimetabolites; AA: alkylating agents; AK: alkaloids; AC: anthracyclines; TX: taxanes; Anti-HER2 agents


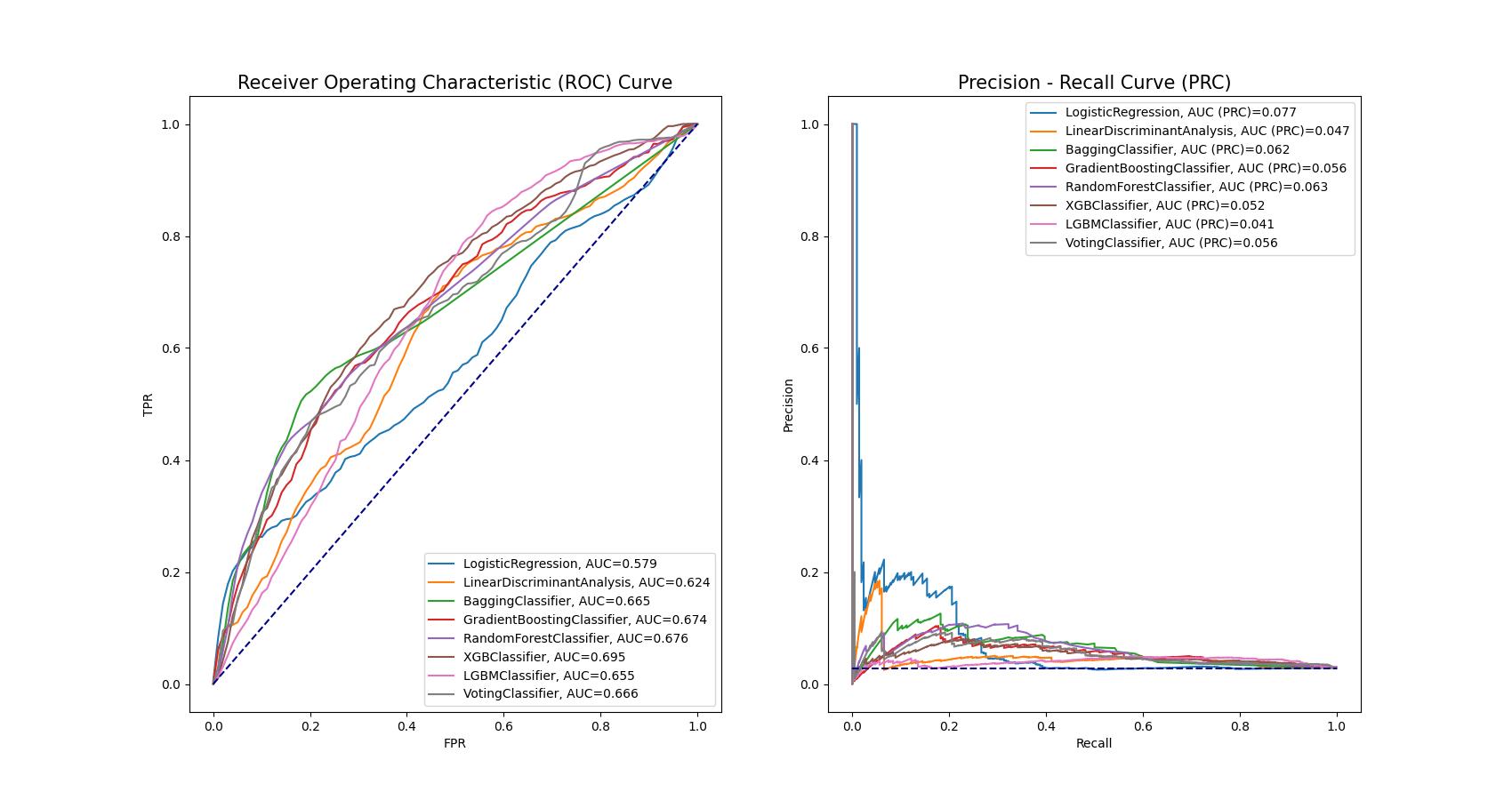


**Figure S2. Model performance with two additional features: laterality and endocrine therapy**
